# A Real-Time Fish Detection System for Partially Dewatered Fish to Support Selective Fish Passage

**DOI:** 10.3390/s25041022

**Published:** 2025-02-09

**Authors:** Jonathan Gregory, Scott M. Miehls, Jesse L. Eickholt, Daniel P. Zielinski

**Affiliations:** 1 Department of Computer Science, Central Michigan University, Mount Pleasant, MI 48859, USA; grego3j@cmich.edu; 2 U.S. Geological Survey, Great Lakes Science Center, Hammond Bay Biological Station, Millersburg, MI 49759, USA; smiehls@usgs.gov; 3 Great Lakes Fishery Commission, Ann Arbor, MI 48105, USA; dzielinski@glfc.org

**Keywords:** selective fish passage, object detection, edge computing, MobileNet SSD, real-time detection

## Abstract

Recent advances in fish transportation technologies and deep machine learning-based fish classification have created an opportunity for real-time, autonomous fish sorting through a selective passage mechanism. This research presents a case study of a novel application that utilizes deep machine learning to detect partially dewatered fish exiting an Archimedes Screw Fish Lift (ASFL). A MobileNet SSD model was trained on images of partially dewatered fish volitionally passing through an ASFL. Then, this model was integrated with a network video recorder to monitor video from the ASFL. Additional models were also trained using images from a similar fish scanning device to test the feasibility of this approach for fish classification. Open source software and edge computing design principles were employed to ensure that the system is capable of fast data processing. The findings from this research demonstrate that such a system integrated with an ASFL can support real-time fish detection. This research contributes to the goal of automated data collection in a selective fish passage system and presents a viable path towards realizing optical fish sorting.

## 1. Introduction

The Laurentian Great Lakes and their tributaries are an extremely important economic and ecological resource for the United States of America and Canada. Not only are the Great Lakes a unique part of the cultural and geographical heritage of North America, but they also support over 1.3 million regional jobs spanning from tourism to shipping that create over USD 82 billion in wages [1]. One particularly important aspect of the Great Lakes is its fisheries, which generate billions of dollars annually from both commercial and recreational fishing [1]. However, threats such as the spread of invasive species and habitat loss degrade the quality of the Great Lakes and their fisheries, making Great Lakes Restoration Initiatives important from both an environmental and an economic standpoint.

The Great Lakes Restoration Initiative (GLRI), a program by the U.S. federal government for investing in protecting and revitalizing the Great Lakes, identifies two focuses for improving the health of the Great Lakes related to fisheries: (1) controlling and eradicating invasive species and (2) improving habitats for native species, particularly by increasing connectivity for these habitats [2]. These two goals can be in conflict, as connecting aquatic habitats that were previously separated by artificial barriers such as dams may have the unintended consequence of allowing non-native species access to pristine habitats [3]. It is therefore necessary for fishery managers to balance the desire for increased connectivity of waterways against the threats posed by non-native or invasive species, a trade-off that has been a subject of much research in recent years [4,5]. Selective connectivity has emerged as a class of physical, chemical, and mechanical methods to support the movement of native fishes around artificial barriers while also preventing uncontrolled passage of non-native species [3,6,7,8]. Thus, selective passage provides a solution to the “connectivity conundrum” of restoring connectivity within and between habitats of native fishes while also preventing the spread of invasive ones to protected waterways [7].

Traditionally, manual trap-and-sort operations have been the only method to guarantee selective passage of desirable fish while blocking undesirable ones. This technique is not scalable and may be harmful to fish populations [7]. Thus, the development of alternative methods to selectively pass fish is needed. At its core, a selective fish passage can be reduced to sort fish according to different behavioral, physiological, phenological, and morphological attributes, a task analogous to the process of single-stream recycling [7]. The success of single-stream recycling is due in part to a shift away from manual sorting to automated processes, a trend that is expected to equally benefit selective fish passages. Automating fish-sorting processes—regardless of which sortable attribute they exploit—should increase the applicability and availability of selective fish passage technologies to waterways across the Great Lakes’ region and beyond.

Sorting fish along morphological differences holds promise for selective passage due to the diversity of body shapes exhibited among fish species. Optical sorting technologies that pair image recognition software with mechanical sorting devices (i.e., operable gates or traps) offer the potential for automated sorting for fish passages. Underwater image-based tools have started to be incorporated into fish-counting devices [9,10]; however, underwater image recognition can be challenging due to poor illumination or high water turbidity [11]. These issues can be avoided if fish are in a partially dewatered state. An Archimedes Screw Fish Lift (ASFL) offers a simple means to capture fish and lift them out of the water for imaging. Pilot tests in a fish crowding structure demonstrated that a prototype ASFL was effective at capturing and safely lifting sucker spp. [12]. Combined with an optical scanner, an ASFL system has the possibility of conducting real-time, accurate fish detection and fish species classification [12].

Image-based fish classification—including classification that distinguishes fish from other objects (single-class) and classification that distinguishes between fish species (multi-class)—is a well-researched domain in the realm of computer vision, with both traditional and deep machine learning approaches being applied to this problem [11]. As summarized in a recent survey [11], traditional machine learning approaches used for fish classification include Naïve Bayesian classification [13,14,15,16], support vector machine classification [17,18,19,20,21,22,23,24,25], and classification by shallow neural networks [26,27,28]. These approaches achieve some success in terms of classification accuracy, but each method requires the data to be preprocessed and input as an engineered feature vector, which is not optimal for robust classification [11].

Recently, deep machine learning has emerged as a popular and effective alternative to these traditional machine learning methods, especially in the realm of image classification. Deep machine learning is distinguished from traditional machine learning in that rather than being given curated feature vectors describing data, deep machine learning models learn a robust representation of the data by mapping inputs to outputs through many layers of a deep neural network [29]. Deep convolutional neural networks (CNNs) are one type of deep machine learning architecture that uses mathematical filters to learn size- and location-invariant representations of objects in images, making CNNs useful for image classification and object detection [29].

Many variations of well-known deep CNN architectures such as AlexNet [30], DenseNet [31], Inception [32], MobileNet [33], ResNet [34], and VGG [35] have been applied to the tasks of fish identification and fish species classification. Examples of these applications include training or fine-tuning versions of these architectures for the task of fish species classification [36,37,38,39] and modifying these architectures to create new deep machine learning fish classifiers [40,41,42,43,44,45,46,47,48,49,50,51,52,53]. Other researchers developed custom deep CNN architectures for fish classification [54,55,56]. Many of these deep CNN classifiers achieved strong classification accuracy, indicating the power and utility of these model architectures.

Fish classification is useful in controlled settings where it is only necessary to identify a single fish in an image. For situations where multiple fish are likely to be present or where the localization of the fish in the image is needed, it is best to use object detection algorithms. Object detection combines object identification and localization by classifying objects within an image and drawing a bounding box around individual object instances [57]. As with fish classification, many different object detection models have been applied extensively to the problem of fish detection. Commonly used models include the You Only Look Once (YOLO) [58] family of single-shot object detectors—especially the YOLOv3 [59], YOLOv4 [60], YOLOv5 [61], and YOLOv7 [62] model architectures—variants of the R-CNN two-stage object detector [63], and various models built using a Single-Shot Multibox Detector (SSD) [64]. Versions of these architectures have been applied to the following domains: single-class [65,66,67,68] and multi-class fish detection [69,70,71,72,73]; fish detection using marine [68,69,72,74] and aquatic [65,66,70,71,75] fish datasets; and detection of both watered [65,66,67,68,71,72,76] and fully or partially dewatered fish [70,77,78]. Although issues related to image quality and class imbalance were common themes in these studies, the abundance of high-performing fish detection models that operate on different domains demonstrates the power and applicability of these deep machine learning architectures to tasks such as fine-grained optical fish sorting and continuous data collection.

Far less research has been applied to using computer vision and deep machine learning algorithms in a selective passage system. Garavelli et al. [79] used an optical sorting system to control the passage of salmon based on fish size as estimated by an image-based algorithm, but this research makes no mention of using deep machine learning to assist with this process. Eickholt et al. [40] demonstrated that a VGG-based deep CNN could classify images of the Great Lakes’ fish species that were partially dewatered and passed through a Whooshh FishL™ Recognition optical scanner with an accuracy of 95%. This deep machine learning classifier could distinguish sea lamprey—an invasive, parasitic fish whose presence in the Great Lakes motivates selective fish passage efforts by the Great Lakes Fishery Commission (GLFC) [80]—from other Great Lakes’ fish with nearly 100% accuracy [40]. However, these authors remarked that this classifier was not tested for real-time fish species classification, and their model was not run using hardware that can be deployed for on-site analysis [40]. Jagadeesan et al. [81] investigated using edge computing to allow for real-time, on-location fish detection to monitor unintended fish passage over a river weir. Edge computing is a computing paradigm where data processing occurs at or close to the site of data collection, allowing for less latency and immediate, automated responses to triggers [82]. Although Jagadeesan et al. did not discuss the applications of their system to fish sorting in a selective passage system, these researchers did present a hardware and software solution using edge computing that could be deployed for various tasks, including fish detection in a selective fish passage system and continuous data collection for fish in situ.

This research seeks to bridge the gap between past studies involving selective fish passage, autonomous fish detection, and edge computing by presenting a case study of a novel and lightweight platform that can support real-time, on-location fish detection for partially dewatered fish exiting a fish lift system. Specifically, this application uses Frigate [83] network video recorder (NVR), a lightweight MobileNetv2 SSD FPN-Lite [84] object detection model trained on a custom dataset, and a Coral Edge TPU USB accelerator to create a fully operational system to support real-time fish lift surveillance. This platform is based on freely available, open-source software and low-cost, commodity hardware. Applications of the platform include monitoring selective fish passage systems, collecting and filtering data from these systems, and alerting fisheries managers about fish-related events. This platform also includes an initial fish species detector that could eventually be used for fine-grained control in a selective fish passage system. This system is built with accessibility, maintainability, and scalability in mind for fishery managers, democratizing AI-assisted fishery surveillance and management even for individuals without specialized knowledge of machine learning. The data, data processing techniques, deep machine learning models, and deployable surveillance platform developed in this research advance progress towards a fully autonomous selective passage system.

## 2. Materials and Methods

### 2.1. Study Site

Video data used in this study were collected as part of a fish passage experiment conducted at the Michigan DNR salmon weir on Swan River, MI, from 23 April to 15 June 2024. Swan River is a small tributary to Lake Huron, near Rogers City, MI. The weir is located approximately 400 m upstream of the river mouth and comprises ten 1.8 m wide flow-through wire mesh screen panels.

### 2.2. Fish Collection

A prototype fish lift consisting of an Archimedes Screw (described previously by Zielinski et al. [12]) was installed at the weir and operated continuously throughout the study period. Fish captured by the lift were raised to approximately 1 m above the water. Upon exit from the upstream end of the ASFL, fish were passed over a dewatering slide made of 12 mm wide aluminum rails spaced 12 mm apart to shed most of the water before being passed through a lighted video chamber. After leaving the video chamber, the fish were captured in a 1.2 m × 1.2 m wire mesh holding cage. Captured fish were removed each morning at approximately 10:00 EST, identified to species, weighed, measured, and then released.

### 2.3. Video Data Collection

**Figure 1:**
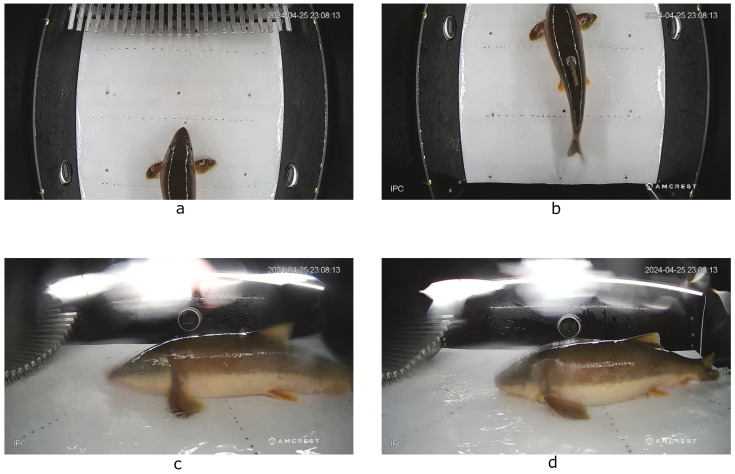
The four camera angles in the ASFL video chamber capturing videos of a fish passing through the chamber. From the top left: Panel (**a**) shows the front overhead camera. Panel (**b**) shows the back overhead camera. Panel (**c**) shows the left camera. Panel (**d**) shows the right camera.

The video chamber was monitored continuously via 4 cameras (8 megapixel, 2.8 mm super wide angle lens, model IP8M-T2599EW manufactured by Amcrest and made in Vietnam) positioned overhead (2 cameras) and at either side. Figure 1 shows the view from each of these four cameras in the video chamber capturing a fish passing through from different angles. Frigate NVR version 0.12.0, a network video recorder used for surveillance and event detection, was used to capture and record the real-time streaming protocol (RTSP) video streams from all four cameras. Frigate ran on a micro factor computer with a Fedora Linux 36 operating system, a 4-core Intel Celeron J4125 CPU, and 8 GB DDR4 memory. Video captured by Frigate was saved to external solid-state hard drives.

### 2.4. Data Curation

Clips of fish passing through the video chamber were extracted from the raw video recordings using heuristic-based video processing. A fast and simple background subtraction script was developed in Python version 3.9.19 using utilities from OpenCV [85] and VidGear [86] to process each video frame in less than 2 milliseconds. Background subtraction is a technique for motion detection where it is assumed that the background in a video sequence changes minimally between frames, therefore allowing moving objects to be detected as deviations from this static environment [87]. A key benefit of the video capture chamber in the ASFL system is that it provides a relatively fixed backdrop, allowing a simple frame differencing approach coupled with a heuristically defined difference threshold to be used for motion detection.

This program initially yielded 120 clips across the four camera angles, resulting from 28 instances of fish passing through the ASFL video capture chamber. Subsequent analysis produced one more fish, contributing to a total of 29 fish across 124 clips. Table 1 shows the details for the fish captured and imaged by the ASFL system. The fish consist of eight species: eight common white suckers (*Catostomus commersonii*), seven steelhead (*Oncorhynchus mykiss*), seven longnose suckers (*Catostomus catostomus*), two largemouth bass (*Micropterus salmoides*), two smallmouth bass (*Micropterus dolomieu*), one rock bass (*Ambloplites rupestris*), one common carp (*Cyprinus carpio*), and one bowfin (*Amia calva*).

**Figure 2:**
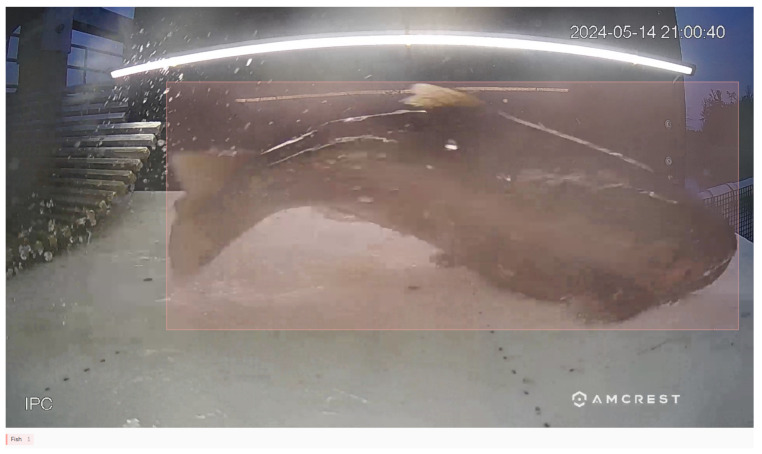
An image containing a fish labeled by hand using Label Studio.

Frames containing a fish (that is, more than just a tail or fin) were extracted from the longer clips, resulting in 4040 images of fish. All frames were saved as 1920 × 1080 images. Initially, the first 1068 images of these fish were annotated by hand for a fish detection task by drawing a bounding box around the fish in the image and labeling the box with the label “Fish”. These 1068 images were experimentally considered a minimal number of fish to annotate that represented a varied sample of different fish passing through the video chamber. This manual annotation was performed using Label Studio [88], an open source data annotation application. Figure 2 shows an example of an image being annotated with this tool. The remaining 2972 images were annotated using a bootstrapping approach in which the initial 1068 annotations were used to train a machine learning annotation tool. This tool then produced annotations for the 2972 unannotated images, which were manually reviewed and re-annotated by hand if any of the annotations contained incorrect bounding boxes.

These data were extended for use in multi-class fish detection tasks by creating separate annotations that used the same bounding boxes as before but contained the individual species labels rather than simply the label “Fish”. Due to the limited number of fish from each species, this dataset from the ASFL best serves the goal of single-class fish detection. However, multi-class fish detection is possible with these data, albeit in a somewhat limited fashion.

For the generic fish detection dataset, denoted as ASFL-Single, only single-class labels (i.e., “Fish”) were used. The ASFL-Single training and independent evaluation data were determined using a random 80/20 split, respectively, of the entire dataset. These two sets are entirely discrete, meaning that no images of fish in the training set exist in the evaluation set. In total, the ASFL-Single training set contains 3276 annotations, and the evaluation set contains 764 annotations. The multi-class fish detection dataset developed for the ASFL system, denoted as ASFL-Multi, used the multi-class labels for each image (i.e., “common white sucker”, “longnose sucker”, etc.). Common carp, bowfin, and rock bass—all species with fewer than two unique fish—were removed from this dataset. For all remaining species of fish, each fish was randomly placed in the training or evaluation set according to an 80/20 split, with at least one fish from each species reserved for the test set. The ASFL-Multi training set contains 3183 annotations, and the evaluation set contains 594 annotations.

The ASFL-Multi dataset suffers from an insufficient number of unique fish from different species. Moreover, it lacks any examples of highly invasive species like sea lamprey (*Petromyzon marinus*) and bighead carp (*Hypophthalmichthys nobilis*). To further study the viability of using object detection for selective passage tasks, an additional dataset of partially dewatered fish from the USGS was curated. This USGS dataset is derived from images of fish passing through a Whooshh FishL™ Recognition optical scanner and contains numerous fish from a similar domain as the ASFL data [40,89]. This dataset contains over 5000 fish across 22 different fish species, including sea lamprey and invasive carp species [89]. It should be noted that all invasive carp in this dataset were culled and then manually passed through the Whooshh scanner, whereas other species were alive while passing through the scanner. Further, all live fish captured by this scanner were manually introduced, rather than volitionally entering the scanning chamber [40]. This manual intervention suggests that the fish in this dataset may have behaved differently from the fish in the ASFL dataset, which did not experience human intervention.

Fish in this USGS dataset were matched to their species labels and annotated with bounding boxes using a machine learning annotation tool bootstrapped with data from the ASFL-Single dataset. All annotations were manually reviewed, and unfit annotations were removed from the dataset. Species with fewer than 5 unique fish were also removed from the data. This process yielded a total of 9510 annotated images spanning 14 species, as shown in Table 2. These annotations were then randomly split using an 80/20 training and evaluation split. As before, special care was taken to ensure that the training and evaluation sets contain no overlap between unique fish. The resulting dataset, called USGS-Multi, contains 7611 training annotations and 1899 evaluation annotations. Each set contains at least one unique fish from each of the species listed in Table 2.

One final dataset, Combined-Multi, was created as a combination of the ASFL-Multi and USGS-Multi training and evaluation subsets. Since common carp are represented in the USGS-Multi training data, the frames of the single common carp from the ASFL data were added to the Combined-Multi evaluation data. Additionally, to mitigate data imbalance, only one sample of each individual fish from the USGS-Multi dataset was added to the training and evaluation sets of Combined-Multi. In total, Combined-Multi contains 7254 training samples and 1357 testing samples. A table summarizing the four datasets created in this research is given in Table 3.

### 2.5. Development Environment

The development environment used for model training and testing was a Len ovo Legion 5 Pro with an AMD Ryzen 7 5800h CPU, an NVIDIA RTX 3070 Mobile GPU with 8 GB of video RAM, and 32 GB of RAM. This system used Ubuntu 22.04.5 as an operating system with Python version 3.9, TensorFlow [90] version 2.7, and CUDA version 12.4 installed. The TensorFlow Object Detection API [91] was used to facilitate model training, fine-tuning, and evaluation.

### 2.6. Model Construction

The base model architecture used in this research for lightweight detection was a MobileNetv2 SSD FPN-Lite 320 × 320 model (hereafter MobileNet SSD), which is supported by the TensorFlow Object Detection API for fast model prototyping and evaluation. MobileNet SSD achieves a benchmark of 22.2% mean average precision and can perform inference in 22 ms, which is equivalent to over 45 frames per second, making this model faster than real time [92]. Moreover, the MobileNet SSD 320 × 320 model is easily configurable to run on the Frigate NVR system using a Coral tensor processing unit (TPU) for edge processing, making this model applicable for edge deployment scenarios. Newer object detection architectures exist, but MobileNet SSD fits the criteria set forth in this research that all components must be open source, easily modifiable, and integrable with minimal modifications with the other components of the fish capture and detection system—namely the Frigate NVR and Coral TPU. It is important when discussing model selection that this research is primarily a case study showing the utility of this fish surveillance system, so this system must be examined as a whole, rather than a sum of parts. Other object detection architectures that are compatible with Frigate and the Coral TPU may be used, but MobileNet SSD is an effective “turnkey” object detection model for this proposed system, leading to its incorporation in this research.

The MobileNet SSD models trained in this research were configured with the same configuration and hyperparameters as the base MobileNetv2 SSD FPN-Lite 320 × 320 model from TensorFlow, which was pretrained on 320 × 320 images from the COCO 2017 dataset [84]. Each model trained in this research was fine-tuned from the base model’s detection checkpoint. This fine-tuning approach greatly reduced the amount of time and data necessary to train robust MobileNet SSD models.

All MobileNet SSD models in this study used momentum stochastic gradient descent (momentum SGD) as an optimizer with cosine decay learning rate scheduling, starting at a base learning rate of 0.08. This learning rate scheduler ensured that as training progressed, increasingly smaller updates were applied to the model, thus helping to mitigate the threat of the model overfitting over many training epochs. The classification loss used was sigmoid focal loss with parameters α=0.25 and γ=2.0, and the localization loss used was smooth L1 loss. Both the box predictor and the underlying feature extractor used ReLU_6 activation functions. Lastly, all models were configured to train using the data augmentation strategies of random horizontal flipping and random cropping. Data augmentation regularizes a model and allows it to generalize better to unknown data by applying image transformation techniques to the training data. This generates new data that are in the same domain as the training data but have markedly different features. This also helps prevent overfitting, which is where a model learns to map its training data perfectly to the training labels but loses the ability to generalize to unseen data.

### 2.7. Model Training

Four distinct models, termed ASFL-Single-Detect, ASFL-Multi-Detect, USGS-Multi-Detect, and Combined-Multi-Detect, were trained, with each one corresponding to one of the four datasets developed for this work. All models used 320 × 320 input sizes, meaning that images were resized during training to 320 × 320 before being sent through the detector. Loss metrics for the training data were logged to monitor the training progress. Moreover, validation data, a subset of the training data on which the model does not train, were evaluated at every 1000 epochs to monitor each model’s performance on new data and track signs of overfitting. The number of epochs on which to train each of the four models was determined by monitoring each model’s performance on these validation data. Owing to both the cosine decay learning rate scheduler and the carefully monitored loss data, no signs of overfitting were detected.

### 2.8. Model Evaluation

Model evaluation was performed by running inference (i.e., generating predictions) on each model’s respective independent evaluation dataset. In all cases, 0.4 was used as the confidence threshold by which a model would generate a prediction. The pycocotools version 2.0.7 Python library from the COCO API [93] was then utilized to get statistics on each trained model’s detection capabilities. Specifically, mAP50, or the mean average precision at an Intersection over Union of 50%, was used in this research as the main evaluation metric for object detection tasks.

Traditional precision and recall classification statistics, which only consider class labels and ignore the fitness of bounding boxes, were also calculated for every class in each model. In this research, these measurements give an indication of how well each model performs in classifying fish, which can be useful apart from bounding box detection. It is also useful to see how many predictions for each class were incorrectly matched to other classes. A confusion matrix succinctly presents these classification statistics in an n×n matrix, where *n* is the number of labels. Suppose the *i*th label in a dataset is indexed as label_*i*_, where *i* is an integer in the range [1,n]. Entries on the *i*th data row of a confusion matrix correspond to images with the ground truth label of label_*i*_, and entries in the *j*th (j∈[1,n]) column of the matrix are images that were predicted with the label of label_*j*_. Table 4 shows an example confusion matrix for a three-class problem, with two added columns showing the precision and recall for each row. The format of Table 4 will be used in the following results to present these classification statistics.

After the initial metric-based evaluation, these models were adapted to run in an edge processing environment to determine their suitability for real-world deployment. Frigate NVR, running on a micro factor computer similar to the one used for data collection, was used as the video processing platform for this evaluation. A Coral Edge TPU USB Accelerator—a small but powerful tensor processing accelerator—allowed these fish detection models to run in real time in the Frigate application. In the absence of a live stream of the ASFL video capture chamber, Fake-RTSP-Streamer [94], a video streaming simulator, was used to simulate a RTSP video stream from an IP camera. To allow the models trained in this research to run on the Coral TPU, every model was converted to a quantized tflite model with 8-bit unsigned integer weights using post-training quantization. All quantized models were then compiled to run on the Coral Edge TPU, which uses a specialized, limited operation set. Since post-training quantization with 8-bit weights may increase a model’s speed at the cost of its performance, the mAP, precision, and recall metrics were again computed after this process to gather accurate data on how these models will perform on this edge system. The speed versus accuracy trade-offs of 8-bit integer quantization are important to consider based on the specific use cases of the model and the requirements of fishery managers. However, at least for the case of high-speed fish detection, speed was deemed a priority in this case study.

Two of these quantized models, ASFL-Single-DetectQ and Combined-Multi-DetectQ, were then tested on this simulated edge system using 7 h of streamed clips from each model’s respective testing data sourced from the back overhead camera angle. Each video hour contained at least one fish from each model’s evaluation set and possibly contained other fish from the training sets. Frigate enables clips from a live RTSP stream containing detections to be saved to the system, so this option was enabled to assess model performance in this simulated deployed setting. The metrics used for this test were precision and recall, where a true positive in this case is a clip of a fish that was successfully saved, a false positive is a clip containing no detections that was saved, and a false negative is a missed detection. Class-specific precision and recall were also calculated where appropriate.

## 3. Results

### 3.1. Model Evaluation

The ASFL-Single-Detect fish detection model was trained for 45,000 epochs. This model achieved a mAP50 score of 0.95 on the evaluation set, as determined by the COCO API mAP50 evaluation tool (Pycocotools version 2.0.7). The full classification confusion matrix for this model is shown in Table 5, where background (BG) indicates all parts of an image that have no annotation. This model achieved a classification precision (Pr) of 1.0 and a recall (R) of 0.96 for the only class, “Fish”.

The next model, ASFL-Multi-Detect, was also trained for 45,000 epochs. The mAP50 score for this model on its evaluation data was 0.20, and the classification statistics shown in Table 6 validate this result.

The third model, USGS-Multi-Detect, was trained for 50,000 epochs, and it achieved a mAP50 score of 0.83 on its evaluation data. The full classification confusion matrix, precision, and recall for this model are all given in Table 7.

The final model, Combined-Multi-Detect, was trained for 50,000 epochs and achieved a mAP50 of 0.47 on its evaluation set. Table 8 shows the classification confusion matrix and precision and recall for this model.

To allow for comparison between Combined-Multi-Detect and ASFL-Multi-Detect (the two models that partially share evaluation data), Combined-Multi-Detect was also evaluated on the evaluation data from ASFL-Multi. Combined-Multi-Detect achieved a mAP50 of 0.31 on these data. Table 9 below shows the classification confusion matrix and precision and recall for Combined-Multi-Detect evaluated on the ASFL-Multi evaluation set.

### 3.2. Quantized Model Evaluation

After each model was quantized and compiled for the Coral TPU, the mAP50 scores, classification confusion matrices, and precision and recall scores for the models were recomputed to account for any altered performance. The quantized ASFL-Single-Detect model, denoted ASFL-Single-DetectQ, was evaluated to have the same mAP50 of 0.95. ASFL-Multi-DetectQ was determined to have a mAP50 of 0.19, a modest loss of 0.01 in mean average precision. USGS-Multi-DetectQ was calculated to have a mAP50 of 0.83, the same mAP50 score as its unquantized counterpart. Finally, Combined-Multi-DetectQ achieved a mAP50 of 0.48 on the Combined-Multi evaluation dataset, a 0.01 increase in mAP. As with mAP50, the confusion matrices and precision and recall scores were similar between the unquantized models and their quantized counterparts. Therefore, for brevity, these tables are omitted but can be made available upon request.

### 3.3. Frigate Evaluation

After quantization evaluation, ASFL-Single-DetectQ and Combined-Multi-DetectQ models were tested independently on the simulated real-time edge environment using Frigate NVR and the Coral Edge TPU. ASFL-Single-DetectQ detected and saved 10 clips, 9 of which contain unique instances of fish. In total, 10 unique fish exist in the video clips on which this model was tested, meaning that ASFL-Single-DetectQ achieved a single-class precision of 0.90 and a recall of 0.90. Combined-Multi-DetectQ detected, classified, and saved 12 clips from its testing video data, 6 of which contain fish. Since fish classification is a factor for this model, an abbreviated confusion matrix for Combined-Multi-DetectQ that contains only fish from the testing videos is given as Table 10. In terms of single-class fish detection, since the ASFL test videos contain 9 unique fish, the Combined-Multi-DetectQ achieved a single-class precision of 0.50 and a recall of 0.67. For multi-class classification precision and recall, refer to Table 10.

## 4. Discussion

### 4.1. Fish Detection Advancements

**Figure 3:**
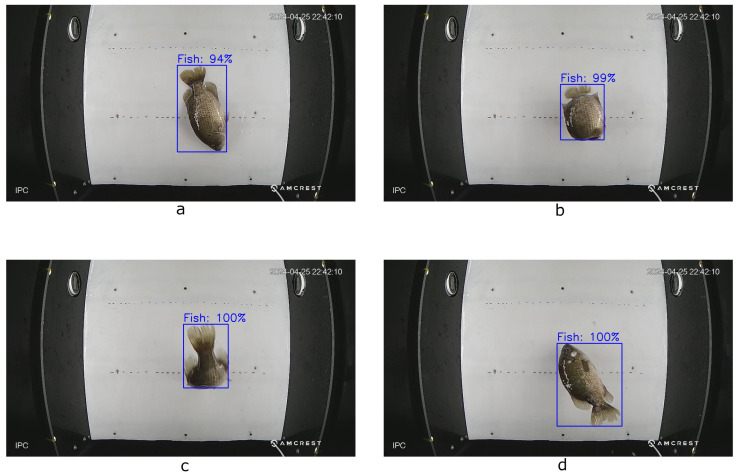
A sequence of ASFL-Single-Detect’s predictions on a rock bass reorienting itself while passing through the ASFL video capture chamber. From the top left: Panel (**a**) shows the fish is initial position. Panel (**b**) shows the fish launching itself into the air. Panel (**c**) shows the fish contorting itself in the air. Panel (**d**) shows the fish landing in the chamber in a new position.

The results from this case study advance efforts for real-time fish detection in multiple ways. First, even with a fairly small dataset composed of only 29 fish, this overall system shows promise in accurately and efficiently detecting fish as they pass through an ASFL-like system. Specifically, the model prototyped for single-class detection in this research, ASFL-Single-Detect, is capable of high-accuracy, generalizable fish detection. Despite the 31 missed detections on frames of fish passing through the video capture chamber, every fish in the ASFL-Single evaluation set was detected partially or fully by ASFL-Single-Detect. In a real-world context, this means that every fish would be recognized and captured by a data capture system, as the redundant frames of the fish passing through the system more than account for occasional missed detections. This claim is supported by the Frigate evaluation of ASFL-Single-DetectQ, which succeeded in detecting and isolating 9 out of the 10 fish in the videos on which it performed inference. This detector is capable of generalizing to predict close-fitting bounding boxes on completely unknown fish in irregular orientations. This is exemplified in Figure 3, which shows the model’s predictions on a rock bass—a fish species that was not represented at all in the model’s training data—as it flips and contorts itself through the ASFL video chamber.

Coupled with its high detection rate and tolerance to quantization, this generablizability indicates that the ASFL-Single-Detect model is well suited for autonomous video chamber monitoring and data collection. This work has shown that this model can seamlessly integrate with Frigate and the Coral Edge TPU for real-time edge data processing, meaning that it can be deployed in situ to assist with real-world fish monitoring. This model has other applications as well. Data collection and processing was the costliest phase of this research in terms of time and effort, and the data culling process using background subtraction still required extensive manual evaluation and labeling. ASFL-Single-Detect can easily automate this process. For example, in the Frigate evaluation, ASFL-Single-Detect generated 10 clips that equated to about 4 min of video from 7 h of a video stream, greatly simplifying the data culling process and reducing manual evaluation to one quick session of reviewing the culled clips. Further, while the model did fail to detect one fish in the Frigate evaluation, this is likely caused by Frigate’s configuration, which uses its own background subtraction algorithm, frame sampling rate, and bounding box selection criteria to filter frames and decide which ones will be sent to the model for evaluation on the Coral TPU. ASFL-Single-Detect could be deployed directly to and outside of Frigate to detect fish in frames, save these frames and their associated clips, and partially annotate these images, all in one step. This autonomous data processing would prove immensely useful in collecting more fish data that can be used to train more robust models in the future.

An important conclusion from these results with the single-class fish detector is that this entire system prototyped for data collection and analysis—the fish detection model, open source NVR, and commodity hardware combined with the ASFL system’s lift, dewatering sled, and video chamber—is viable and eligible for immediate deployment. By following this case study’s experimental setup, fishery managers can replicate these results with their own data gathered in situ and build a similar surveillance platform without costly software and hardware requirements. This proof of concept in a democratized, user-friendly, and readily deployable fish collection and surveillance system has major implications for advancing fishery management and increasing the tools available for fishery managers to incorporate artificial intelligence into their management efforts. Further, by decoupling the surveillance aspects of this system from the physical ASFL device, this system can be extended beyond fishery management into other realms where it is necessary to rapidly collect data, train models, and deploy hardware for active surveillance tasks.

**Figure 4:**
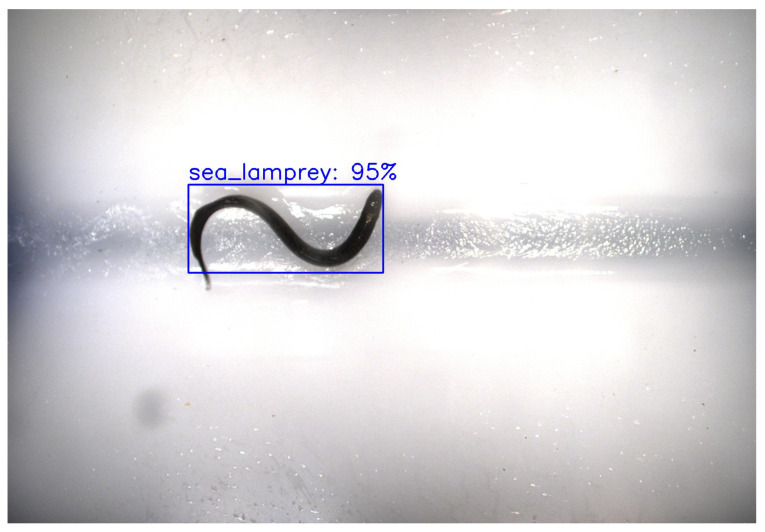
An image containing a sea lamprey successfully predicted by USGS-Multi-Detect.

Second, combining the ASFL data with similar data sources was shown to be a successful way to increase model performance on multi-class fish detection despite obvious data limitations. As expected, the ASFL dataset suffers from class imbalance problems that decrease the effectiveness of ASFL-Multi-Detect for multi-class fish detection. For example, images of a singular longnose sucker that lingered in the ASFL video capture chamber make up roughly 38% of the training data. As a result, the model achieves a low mAP50 of 0.20, which would be unsuitable for real-world applications where recognizing fish is key to preventing the spread of invasive species. Moreover, the ASFL-Multi-Detect model cannot be used for any sort of invasive species detection or control, as its training data contain no examples of sea lamprey, invasive carp, or other invasive species. However, as demonstrated by USGS-Multi-Detect, which achieved a mAP50 of 0.83, a MobileNet SSD model trained on varied data representing many unique fish can lead to very accurate results, including the detection of invasive species like sea lamprey and invasive silver carp with the very high recall values of 0.95 and 0.99, respectively (see Table 7). Figure 4 shows an example of an invasive sea lamprey being successfully localized and classified by the USGS-Multi-Detect model. This suggests that with more and better data, ASFL-Multi-Detect may be capable of reaching a similar level of success.

The most interesting results occur when the ASFL and USGS datasets are combined. The data from the USGS dataset do not generalize well to the data from the ASFL and vice versa. However, by immunizing the dataset with enough samples from each dataset, a model trained on this combined dataset can learn a more robust representation of the classes that are shared between these two data sources. As presented in the results, when performing inference on the evaluation data from ASFL-Multi (which is a proper subset of the evaluation data for Combined-Multi), Combined-Multi-Detect achieved a mAP50 of 0.31, which is 0.11 greater than the mAP50 score for ASFL-Multi-Detect. Further, compare Table 6, the classification confusion matrix for ASFL-Multi-Detect, with Table 9, the classification confusion matrix for Combined-Multi-Detect evaluated on the ASFL-Multi evaluation set. Almost every category in Table 9 outperforms its counterpart in Table 6, especially in terms of improving recall. This demonstrates that more data, even from a different data source, are helpful in increasing the model’s performance on a target domain.

As evidenced by the Frigate evaluation, Combined-Multi-Detect is not yet strong enough to be used for pure multi-class detection tasks, as it is too prone to fish misclassifications, false negatives, and false positives that could prove disastrous in a selective passage system. Further, although this model is trained on images of invasive species passing through the Whooshh scanning system, it is untested whether this model could accurately identify invasive species passing through the ASFL system, in which fish enter volitionally, have far more agency, and move with more explosive action than in the Whooshh system. Still, as a data-gathering tool, the Combined-Multi-Detect model certainly has a utility. Moreover, this model serves as a proof of concept that (1) high-performance fish species classification is possible using images of partially dewatered fish passing through an ASFL system and that (2) combining datasets from similar detection domains is an inexpensive and effective way to bolster model performance.

It is also worthwhile to note that the fish passing through the video chamber in the ASFL system were allowed a full range of movement, meaning that for some frames captured of fish moving through the ASFL from different angles, the presentation of the fish did not contain distinguishing features. All models were evaluated on a frame-by-frame basis, meaning that the model inaccuracies may be due in part to these poor fish presentations. Therefore, a majority voting approach where a single fish passing through the ASFL is labeled according to the most prevalent predicted label across all frames of the fish captured from multiple camera angles could be utilized to improve this system’s multi-class detection capabilities. Using such an approach, the overall classification accuracy of ASFL-Multi-Detect, for example, would be 50% (four out of eight unique fish) even with the current data limits. A composite image approach similar to the one used by [40] could also be used to train models with more details about a fish passing through the ASFL. However, both of these methods would complicate both the real-time requirements of this system and the model’s integrability with Frigate. These paths for improvement are left open, but given the current system’s emphasis on deployability and simplicity, these techniques were not implemented in this case study.

### 4.2. Real-Time Processing

The edge processing environment used in this research demonstrated its effectiveness as a lightweight and open source solution to selective passage monitoring and, eventually, control. With the exception of ASFL-Multi-DetectQ, the models trained in this research suffered very few performance losses as a result of being quantized and compiled to run on the Coral TPU in this environment, suggesting that most models will not suffer when they are ported onto this system. In fact, quantization appeared to boost the performance of some models, such as Combined-Multi-DetectQ, which increased its mAP50 by 0.01 after quantization. Detection on this system takes 22 ms, which is equivalent to just over 45 fps, making this system capable of real-time detection for most video feeds. This system is resource-efficient, extremely portable, capable of offline operation, and easy to configure and manage for individuals who do not have a background in software engineering. Importantly, this system can be deployed immediately using readily available commodity hardware to similar systems that use RTSP-streamed video to monitor partially dewatered fish. Although models used in custom deployments may need to be fine-tuned on specific camera angles and views, the training, evaluation, and deployment pipeline presented in this research simplifies this process, enabling fishery managers to gain more control over utilizing these technologies in emergent use cases.

### 4.3. Research Limitations

One limitation in this study is the lack of data from different environmental conditions that adequately represent every fish species that is required for robust selective passage monitoring and control. As mentioned before, this system remains untested on tasks like classifying sea lamprey passing through the ASFL. Moreover, while the generalization capabilities of the single-class and multi-class detection models appear to be robust, only a deployment using realistic data can adequately assess this. As such, the models developed in this research should only be used for data collection and annotation. However, even then, a human-in-the-loop approach is necessary to validate model predictions and continuously improve model performance by training the model on newly discovered data. The results from this case study indicate that an iterative, bootstrapping approach for training increasingly better models is viable in this system, meaning that these limitations will subside with more experimental data collection. Further, these limitations are with the models and not the system as a whole—that is, if comprehensive data for a specific fishery surveillance task are available, this case study suggests that this surveillance platform will perform well.

### 4.4. Future Work

This research presents several intriguing paths for future work. First, using the models developed in this research, more data should be continuously collected from ASFL systems, annotated, and used to train new machine learning models that have a greater exposure to different species of fish, variations in lighting, and other noisy environmental conditions. Second, the close-fitting bounding boxes generated by the models in the controlled ASFL scanning environment open the possibility to developing additional software to predict and record the lengths, girths, and weights of fish passing through the ASFL. Ideally, the models developed in this research can be used not just to detect fish but eventually record fish measurements as well, extending the ASFL system to be used for autonomous fishery health monitoring in situ. Third, future research should carry out an investigation using larger, more powerful open source models than MobileNetv2 SSD FPN-Lite 320 × 320. MobileNetv2 SSD FPN-Lite 320 × 320 was chosen in this research due to its highly configurable nature, simple training pipeline, and compatibility with Frigate and the Coral TPU, which makes this model accessible for use by individuals without a background in machine learning. However, for the best model performance, newer object detection architectures should be tested and adapted for use in Frigate or used on their own in custom network video recording applications. Finally, the multi-class detection results pose the question of whether species-level classification is necessary, or if classifying similar fish based on general characteristics would be sufficient. For example, grouping common white and longnose suckers together—fish that are quite similar in appearance and are both native to the Great Lakes—would likely increase the mAP of the models in this research if the data are clear. Using the training pipeline and data collection paradigm presented in this research, fishery managers can create specific selective passage datasets on a case-by-case basis, granting a high degree of flexibility in multi-class fish detection.

The most exciting avenues for this research are other deployments of this system in waterways across the Great Lakes’ region and beyond. As mentioned before, fishery managers can replicate the experiments from this research and deploy similar systems without relying on advanced hardware or software engineering expertise. The open source, accessible nature of the components of this platform contribute to lowering the costs of intelligent fishery surveillance and present a simple, “turnkey” approach for similar systems to be readily deployed in the wild. It is the hope that in the spirit of this research’s open science efforts, other researchers and fishery managers will share their data and results, contributing to a pool of knowledge that can be used to iteratively improve this system as a whole with more data and expertise.

## 5. Conclusions

Selective fish passages, a key component of improving fishery health in the Great Lakes and beyond, can only be made scalable through the development of autonomous fish-sorting techniques. To support this goal, this case study presents an integrated, readily deployable platform for fish surveillance in an ASFL-like system. Using experimental data of partially dewatered fish exiting an Archimedes Screw Fish Lift gathered in situ, several models were developed that will be used on this platform for data collection, annotation, and fishery monitoring. These models were fine-tuned versions of a standard MobileNetv2 SSD FPN-Lite 320 × 320 object detection model and were trained using custom datasets of fish passing through optical scanners for the tasks of single-class and multi-class fish detection. These models were then adapted for deployment in an edge processing environment, and the two most successful models were tested on an edge processing environment for real-time data collection and annotation tasks. The results show that the models developed in this research are best used for single-class fish detection, which can be used to assist in real-time data collection efforts. Multi-class models, while showing promise, must be trained on larger datasets to gain robust detection capabilities. This research also shows that these models are easily deployable for real-time detection on an edge processing system, thus fulfilling a need in fishery monitoring. Most importantly, the components of this system interface flawlessly, providing a definitive proof of concept that this surveillance platform is viable and useful.

Moving forward, this platform will enable the collection of larger and more detailed datasets that can be used to assist with the goal of optical fish sorting in a selective passage system. Future research can build on these results and improve model performance on classifying different fish species, eventually leading to the level of sensitivity necessary for optical fish sorting. As a note of caution, fishery managers must be wary about placing too much faith in machine learning models, which are ultimately black boxes that defy explanation at times. However, as a means to autonomously gather data and impose a software-supported backstop for selective fish passage, this platform demonstrates robust detection capabilities that will only improve as its models are trained on more and better data.

## Figures and Tables

**Table 1:** The time recorded, common name, and scientific name of fish extracted from the ASFL video chamber recordings.

Time Recorded	Common Name	Scientific Name
25 April 2024 16:54	Common White Sucker	*Catostomus commersonii*
25 April 2024 22:42	Rock Bass	*Ambloplites rupestris*
25 April 2024 22:47	Common White Sucker	*Catostomus commersonii*
25 April 2024 23:08	Longnose Sucker	*Catostomus catostomus*
25 April 2024 23:50	Longnose Sucker	*Catostomus catostomus*
26 April 2024 00:58	Common White Sucker	*Catostomus commersonii*
26 April 2024 01:04	Longnose Sucker	*Catostomus catostomus*
26 April 2024 01:13	Longnose Sucker	*Catostomus catostomus*
26 April 2024 01:37	Longnose Sucker	*Catostomus catostomus*
26 April 2024 22:57	Common White Sucker	*Catostomus commersonii*
27 April 2024 00:28	Longnose Sucker	*Catostomus catostomus*
27 April 2024 01:19	Longnose Sucker	*Catostomus catostomus*
27 April 2024 03:46	Common White Sucker	*Catostomus commersonii*
27 April 2024 22:15	Common White Sucker	*Catostomus commersonii*
27 April 2024 23:24	Steelhead Trout	*Oncorhynchus mykiss*
28 April 2024 04:30	Steelhead Trout	*Oncorhynchus mykiss*
29 April 2024 01:01	Common White Sucker	*Catostomus commersonii*
29 April 2024 23:59	Steelhead Trout	*Oncorhynchus mykiss*
30 April 2024 02:49	Steelhead Trout	*Oncorhynchus mykiss*
30 April 2024 18:48	Common Carp	*Cyprinus carpio*
2 May 2024 13:38	Largemouth Bass	*Micropterus salmoides*
2 May 2024 14:12	Largemouth Bass	*Micropterus salmoides*
3 May 2024 02:02	Common White Sucker	*Catostomus commersonii*
3 May 2024 19:59	Smallmouth Bass	*Micropterus dolomieu*
4 May 2024 15:28	Smallmouth Bass	*Micropterus dolomieu*
4 May 2024 21:44	Bowfin	*Amia calva*
10 May 2024 01:55	Steelhead Trout	*Oncorhynchus mykiss*
11 May 2024 04:27	Steelhead Trout	*Oncorhynchus mykiss*
14 May 2024 21:00	Steelhead Trout	*Oncorhynchus mykiss*

**Table 2:** The common names, scientific names, and counts of fish in the USGS-Multi dataset.

Common Name	Scientific Name	Count
Bighead Carp	*Hypophthalmichthys nobilis*	32
Common Carp	*Cyprinus carpio*	102
Common White Sucker	*Catostomus commersonii*	1689
Longnose Gar	*Lepisosteus osseus*	34
Longnose Sucker	*Catostomus catostomus*	1580
Quillback Carpsucker	*Carpiodes cyprinus*	181
Rainbow Trout	*Oncorhynchus mykiss*	676
Redhorse Sucker	*Moxostoma carinatum*	81
Sea Lamprey	*Petromyzon marinus*	1409
Silver Carp	*Hypophthalmichthys molitrix*	1417
Smallmouth Bass	*Micropterus dolomieu*	27
Smallmouth Buffalo	*Ictiobus bubalus*	216
Walleye	*Sander vitreus*	2034
White Bass	*Morone chrysops*	32

**Table 3:** Summary of the fish detection datasets created for this research.

Dataset Name	Source	Task	Number of Species	Number of Annotations in Training Dataset	Number of Annotations in Evaluation Dataset
ASFL-Single	ASFL	Single-Class Detection (i.e., “Fish”)	-	3276	764
ASFL-Multi	ASFL	Multi-Class Detection (i.e., species label)	5	3183	594
USGS-Multi	USGS	Multi-Class Detection	14	7611	1899
Combined-Multi	USGS & ASFL	Multi-Class Detection	15	7254	1357

**Table 4:** An example classification confusion matrix, precision (Pr), and recall (R) for a three-class problem with A, B, and C labels.

True (T)\Predicted (P)	A	B	C	Precision (Pr)	Recall (R)
**A**					
**B**					
**C**					

**Table 5:** The classification confusion matrix, precision (Pr), and recall (R) for ASFL-Single-Detect evaluated on the ASFL-Single evaluation dataset with background (BG) and Fish class labels.

T\P	BG	Fish	Pr	R
**BG**	-		-	-
**Fish**	31	733	1.0	0.96

**Table 6:** The classification confusion matrix, precision (Pr), and recall (R) for ASFL-Multi-Detect evaluated on the ASFL-Multi evaluation dataset with background (BG), common white sucker (CWS), longnose sucker (LNS), smallmouth bass (SMB), steelhead (STL), and largemouth bass (LB) labels.

T\P	BG	CWS	LNS	SMB	STL	LB	Pr	R
**BG**	-						-	-
**CWS**	16	115	4		40		0.39	0.66
**LNS**	14	164	34		38		0.89	0.14
**SMB**	1	10			37		0.00	0.00
**STL**	5	3			53		0.27	0.87
**LB**	19	1			29	11	1.00	0.18

**Table 7:** The classification confusion matrix, precision (Pr), and recall (R) for USGS-Multi-Detect evaluated on the USGS-Multi evaluation dataset with background (BG), common white sucker (CWS), longnose sucker (LNS), smallmouth bass (SMB), steelhead (STL), common carp (CC), bighead carp (BC), longnose gar (LG), quillback carpsucker (QC), redhorse sucker (RS), sea lamprey (SL), silver carp (SC), smallmouth buffalo (SB), walleye (W), and white bass (WB) labels.

T\P	BG	CWS	LNS	SMB	STL	CC	BC	LG	QC	RS	SL	SC	SB	W	WB	Pr	R
**BG**	-															-	-
**CWS**		316	9					3		1				4	1	0.79	0.95
**LNS**	5	57	235											1		0.95	0.79
**SMB**		2		4	2									1		1.00	0.44
**STL**		1	2		148			1								0.99	0.97
**CC**						9										0.90	1.00
**BC**							2					3				0.50	0.40
**LG**								10								0.29	1.00
**QC**	1	1							45	1						1.00	0.94
**RS**		3								3						0.50	0.50
**SL**	1	2	1							1	245			9		1.00	0.95
**SC**	1					1	2					297				0.99	0.99
**SB**		2											44			1.00	0.96
**W**	2	14	1					20						379		0.96	0.91
**WB**															6	0.86	1.00

**Table 8:** The classification confusion matrix, precision (Pr), and recall (R) for Combined-Multi-Detect evaluated on the Combined-Multi evaluation dataset with background (BG), common white sucker (CWS), longnose sucker (LNS), smallmouth bass (SMB), steelhead (STL), largemouth bass (LB), common carp (CC), bighead carp (BC), longnose gar (LG), quillback carpsucker (QC), redhorse sucker (RS), sea lamprey (SL), silver carp (SC), smallmouth buffalo (SB), walleye (W), and white bass (WB) labels.

T\P	BG	CWS	LNS	SMB	STL	LB	CC	BC	LG	QC	RS	SL	SC	SB	W	WB	Pr	R
**BG**	-																-	-
**CWS**	9	231	1		47	1											0.44	0.80
**LNS**	10	200	134		12										1		0.99	0.38
**SMB**	2	9		4	34	2											0.31	0.08
**STL**		4			108												0.47	0.96
**LB**	2	16		8	15	18										1	0.67	0.30
**CC**	10	30			4	6	2										1.00	0.04
**BC**								1					2				1.00	0.33
**LG**		2							2								1.00	0.50
**QC**		1			2					12					1		1.00	0.75
**RS**		2															0.00	0.00
**SL**												127					0.99	1.00
**SC**					1						1		123				0.98	0.98
**SB**		1		1										15			1.00	0.88
**W**	1	34	1		2							1			100		0.98	0.72
**WB**					3												0.00	0.00

**Table 9:** The classification confusion matrix, precision (Pr), and recall (R) for Combined-Multi-Detect evaluated on the ASFL-Multi evaulation set with background (BG), common white sucker (CWS), longnose sucker (LNS), smallmouth bass (SMB), steelhead (STL), and largemouth bass (LB) labels.

T\P	BG	CWS	LNS	SMB	STL	LB	Pr	R
**BG**	-						-	-
**CWS**	9	118			47	1	0.42	0.67
**LNS**	11	137	90		12		1.00	0.36
**SMB**	2	8		4	32	2	0.33	0.08
**STL**		2			59		0.36	0.97
**LB**	3	16		8	15	18	0.86	0.30

**Table 10:** The classification confusion matrix, precision (Pr), and recall (R) for Combined-Multi-DetectQ running in the simulated edge environment on videos containing a subset of its ASFL evaluation data with background (BG), common white sucker (CWS), longnose sucker (LNS), smallmouth bass (SMB), steelhead (STL), largemouth bass (LB), and common carp (CC) labels.

T\P	BG	CWS	LNS	SMB	STL	LB	CC	Pr	R
**BG**	-			1	5			-	-
**CWS**		1						0.25	1.00
**LNS**	1	2						0.00	0.00
**SMB**	1							0.00	0.00
**STL**					2			0.29	1.00
**LB**	1							0.00	0.00
**CC**		1						0.00	0.00

## Data Availability

Datasets used in this study are available at https://doi.org/10.17605/OSF.IO/QTRKD. The code used in this project can be made available upon request.
